# The F_1_F_o_-ATP synthase α subunit of *Candida albicans* induces inflammatory responses by controlling amino acid catabolism

**DOI:** 10.1080/21505594.2023.2190645

**Published:** 2023-03-29

**Authors:** Shuixiu Li, Yuting Liu, Luobei Weng, Yajing Zhao, Yishan Zhang, Zhanpeng Zhang, Yang Yang, Qiaoxin Chen, Xiaocong Liu, Hong Zhang

**Affiliations:** a Department of Dermatology, The First Affiliated Hospital of Jinan University, Guangzhou, Guangdong, China; b Institute of Mycology, Jinan University, Guangzhou, Guangdong, China

**Keywords:** Candida albicans, F1fo-ATP synthase, α subunit, inflammatory response, amino acid catabolism

## Abstract

Sepsis is a leading cause of fatality in invasive candidiasis. The magnitude of the inflammatory response is a determinant of sepsis outcomes, and inflammatory cytokine imbalances are central to the pathophysiological processes. We previously demonstrated that a *Candida albicans* F_1_F_o_-ATP synthase α subunit deletion mutant was nonlethal to mice. Here, the potential effects of the F_1_F_o_-ATP synthase α subunit on host inflammatory responses and the mechanism were studied. Compared with wild-type strain, the F_1_F_o_-ATP synthase α subunit deletion mutant failed to induce inflammatory responses in *Galleria mellonella* and murine systemic candidiasis models and significantly decreased the mRNA levels of the proinflammatory cytokines IL-1β, IL-6 and increased those of the anti-inflammatory cytokine IL-4 in the kidney. During *C. albicans*-macrophage co-culture, the F_1_F_o_-ATP synthase α subunit deletion mutant was trapped inside macrophages in yeast form, and its filamentation, a key factor in inducing inflammatory responses, was inhibited. In the macrophage-mimicking microenvironment, the F_1_F_o_-ATP synthase α subunit deletion mutant blocked the cAMP/PKA pathway, the core filamentation-regulating pathway, because it failed to alkalinize environment by catabolizing amino acids, an important alternative carbon source inside macrophages. The mutant downregulated Put1 and Put2, two essential amino acid catabolic enzymes, possibly due to severely impaired oxidative phosphorylation. Our findings reveal that the *C. albicans* F_1_F_o_-ATP synthase α subunit induces host inflammatory responses by controlling its own amino acid catabolism and it is significant to find drugs that inhibit F_1_F_o_-ATP synthase α subunit activity to control the induction of host inflammatory responses.

## Introduction

The morbidity and mortality rates of invasive candidiasis (IC) have remained high in immunocompromised individuals [1] and are 7.07/1000 [2] and 42.0%–79.9% [2,3], respectively. Sepsis is a major reason of fatality in IC [4,5], resulting in a mortality rate of 66% [2].

The magnitude of the inflammatory response is a determinant of sepsis outcomes and imbalances in inflammatory cytokines are central to the pathophysiological processes [6]. When *Candida albicans* invades the host, filamentation triggers the discharge of proinflammatory cytokines interleukin (IL)-1β [7–10] and IL-6 [11], leading to host inflammatory responses. The systemic hyperinflammatory response is a main characteristic of the early stages of sepsis [6,12]. During this period, the massive rapid secretion of proinflammatory cytokines, including IL-1β, IL-6, IL-12 and IL-23 [6,13], exacerbate the systemic inflammatory response and then cause multiorgan damages in severe cases [14]—especially to renal failure [15–17], and eventually promote the progression of sepsis to irreversible septic shock [6,13,18]. In addition, the discharge of anti-inflammatory cytokines IL-4 [19] or IL-10 [12,20,21] can control the progression of sepsis.

Macrophages are critical for controlling host inflammatory responses by phagocytizing and killing *C. albicans* [14,22]. Preventing *C. albicans* from escaping macrophages effectively alleviates host inflammatory responses by reducing the excessive release of IL-1β [14]. Macrophages kill and eliminate phagocytized *C. albicans* mainly through intracellular mechanisms of nutrient restriction and acidic phagosomes [23]. However, amino acids, which are abundant inside macrophages are an important alternative carbon source for the immune evasion of *C. albicans* [24–28]. Phagocytized *C. albicans* catabolizes amino acids to produce NH_3_ to neutralize the acidity of phagosomes [26,27], which induces filamentation for macrophage killing and escape [26,29], thus leading to host inflammatory responses [14]. The cAMP/PKA signalling pathway plays a core role in regulating the filamentation of *C. albicans* [30], and pH is one of the upstream signalling molecules of this pathway [31]. Therefore, the regulatory mechanism by which *C. albicans* alkalinizes environment to induce filamentation may be related to pH signalling.

Proline dehydrogenase (Put1) and ∆^1^-pyrroline-5-carboxylate dehydrogenase (Put2), localized in mitochondria, are two key enzymes essential for amino acid catabolism in *C. albicans* to produce NH_3_ to alkalinize environment [32,33]. Furthermore, the functional electron transport chain (ETC) involved in oxidative phosphorylation (OXPHOS) [34] is required for Put1 activity [35,36]. And the F_1_F_o_-ATP synthase at the terminal of ETC is a multisubunit enzyme with dual functions of synthesizing and hydrolysing ATP [37]. Although we previously demonstrated that a *C. albicans* strain with deficiency of the α subunit, an important subunit of F_1_F_o_-ATP synthase, was nonlethal to mice [38], the role of this subunit in the host septic hyperinflammatory response and its underlying molecular mechanism remains unknown. It is speculated that F_1_F_o_-ATP synthase α subunit deletion may prevent the induction of host inflammatory responses at the source by inhibiting amino acid catabolism in *C. albicans*. Therefore, to clarify the effect of the F_1_F_o_-ATP synthase α subunit on host inflammatory responses and its mechanism, the infection model in *Galleria mellonella*, the bloodstream infection model in mice, *C. albicans*-macrophage co-culture and macrophage-mimicking microenvironment were established in this study, and the indexes of host inflammatory responses, inflammatory cytokines, macrophage interactions, filamentation, the cAMP/PKA pathway, the signalling molecule pH, amino acid catabolism and OXPHOS were evaluated in the F_1_F_o_-ATP synthase α subunit deletion strain (*atp1*∆/∆) and its parent strain (wild-type, WT).

This study demonstrated that the *C. albicans* F_1_F_o_-ATP synthase α subunit induces host inflammatory responses by controlling its own amino acid catabolism from the host, cellular and molecular levels through a series of interlinked and gradually in-depth of experiments. Therefore, it is significant to find drugs that inhibit the activity of this subunit to control the induction of host inflammatory responses.

## Methods

### Strains and culture media

A strain of deletion of the F_1_F_o_-ATP synthase α subunit-encoding gene *ATP1* (*atp1*Δ/Δ) [38] and its parent strain *C. albicans* SC5314 (WT) [39] were used in this study. And they were activated on yeast extract peptone glucose (YPD, composed of 1% yeast extract, 2% peptone and 2% glucose, supplemented with (solid medium) or without (liquid medium) 2% agar) solid medium and then cultured overnight in YPD liquid medium before being used in the experiments [38]. To simulate the reported acidic [23] and amino acid-rich [24,25] microenvironment of macrophages, the macrophage-mimicking medium was composed of 0.17% yeast nitrogen base (YNB, without amino acids and with 0.5% ammonium sulphate) [26,27,33], 2% casamino acids (CAA, a casein hydrolysate containing 18 kinds of amino acids required by *C. albicans*) [26,27] and 0.2% glucose [32] (low concentration of glucose is present in early phagosomes of macrophages [23]), supplemented with (solid medium) or without (liquid medium) 2% agar, and the pH of the medium was adjusted to acidic pH 5.0 (the pH of acidic phagosomes of macrophages is 4.7 ~ 5.5 [23]) using 1 M HCl [26]. In order to further investigate the amino acid catabolism of the above strains, the CAA medium [32] containing 0.17% YNB and 2% CAA was used, supplemented with (solid medium) or without (liquid medium) 2% agar.

### Effect of the F_1_F_o_-ATP synthase α subunit on the inflammatory response of G. mellonella

*G. mellonella* is a well-established model to study host immune responses induced by *C. albicans* [40,41]. Six-week-old larvae of *G. mellonella* were stored in wood chips at 15 °C in the dark before use. Strains were incubated in YPD liquid medium for 12–16 h, then resuspended with normal saline (NS, 0.9% sterile sodium chloride injection). A lethal dose of WT (5 × 10^5^ colony-forming units (CFU)) [42] or *atp1*∆/∆ (5 × 10^5^ CFU) or 100 μl NS was injected into the haemocoel of larvae through the last proleg. Then, the larvae were incubated in a breathable plastic box in the dark at 37 °C. The number of surviving larvae was counted at intervals. Larvae that did not show any signs of movement upon contact were considered dead. The percentage of surviving larvae was calculated, and the data were plotted on a survival curve. For histopathologic analysis to observe tissue inflammatory responses, at 72 h after infection, three larvae from each group were fixed, embedded, sectioned and finally stained with periodic acid-Schiff (PAS). All assays were repeated three or more times.

### MicroPET/CT and biodistribution assays

This study used female BALB/c mice (8–10 weeks, 18–22 g). The strains were incubated in YPD liquid medium for 12–16 h, then washed and resuspended with NS. Seventy-two hours after intravenous injection of a lethal dose of WT (5 × 10^5^ CFU) [43] or *atp1*∆/∆ (5 × 10^5^ CFU) or 100 μl NS, three mice in each group were intraperitoneally injected with 1% pentobarbital at 15 μl/g, then intravenously injected with [^18^F]FDG (2-[^18^F]fluoro-2deoxy-D-glucose, HTA) and scanned with a microPET/CT scanner (Instrument). The radioactivity accumulation was represented as a ratio of [^18^F]FDG dose per gram (% ID/g). For *ex vivo* biodistribution assay, the mice were humanely sacrificed and organs were harvested and weighed. A γ-counter was used to determine the radioactivity of the organs and the biodistribution was represented as a ratio of [^18^F]FDG dose per gram (% ID/g). All assays were repeated three or more times.

### Histopathologic analysis

Seventy-two hours after intravenous infection, three mice from each group were sacrificed. Then, the kidneys were harvested aseptically, fixed, embedded, sectioned and finally stained with PAS. According to the approval of the Experimental Animal Ethics Committee of Jinan University, the execution process of all animal trials in this study followed the standards of the experimental animal ethical commitment letter. All assays were repeated three or more times.

### Confocal laser scanning microscopy (CLSM)

RAW264.7 macrophages (ATCC TIB-71) were pretreated with Mitotracker® Deep Red FM (Invitrogen) for 15–45 min and the cells of *C. albicans* strains were pretreated using fluorescein isothiocyanate (FITC, Sigma) for 10–30 min. After 3 h of 1:1 co-culture, images were taken with CLSM (Carl Zeiss, LSM880) and analysed using ZEN 2.3 software. The proportion of hyphal cells inside macrophages was counted using ImageJ software from at least 50 cells of each strain. All assays were repeated three or more times.

### Real-time quantitative polymerase chain reaction (Rt‐qpcr)

Total RNA of mouse kidney tissue homogenates was obtained by TRIzol (Invitrogen) [44]. Total RNA of the strains was obtained by a fungal RNA extraction Kit (Omega Bio-Tek) [38]. The cDNA obtained after reverse transcription of total RNA was used in RT-qPCR. GAPDH and 18S rRNA acted as housekeeping genes in mice and *C. albicans*, respectively. The 2^−ΔΔCT^ method was carried out to obtain the mRNA expression level. Supplementary Table S1 exhibits all primers used. Assays were repeated three or more times.

### Protein kinase a (PKA) activity, cyclic adenosine monophosphate (cAMP) content, and rat sarcoma 1 (Ras1) activity assays

Before collected, the strains in macrophage-mimicking liquid medium (pH 5; 0.17% YNB+2% CAA+0.2% glucose) [23,26] or CAA liquid medium (0.17% YNB+2% CAA) [32] were incubated at 37°C for 12 h. Firstly, to assess the PKA activity, total proteins obtained by liquid nitrogen grinding were detected by a PKA activity assay kit (Promega, V5340, USA) and the gel was observed under UV light to evaluate the peptides of phosphorylated PKA and non-phosphorylated PKA by comparing them with the positive (+) and negative (-) control wells. Second, to assess the intracellular cAMP content, the cells were mixed and incubated with reagents from the cAMP-Glo™ Assay Kit (Promega, V1501), and then chemiluminescence (in RLU) of the samples was determined by a multifunctional microplate reader. The intracellular cAMP content in each strain was calculated as ΔRLU = RLU (control well) – RLU (sample well), and the cAMP content (in nM) was obtained by substituting the RLU values into the standard curve equation. Finally, to assess the Ras1 activity (GTP-Ras1), total protein was obtained by liquid nitrogen grinding, then active Ras1 was obtained from total protein using an Active Ras pull-down assay kit (Pierce, 16117). Subsequently, total protein and active Ras1 were subjected to immunoblotting assay. The first antibody was an anti-Ras clone antibody (1:1000; Millipore, 05–516), and the second antibody was a goat anti-mouse secondary antibody (1:10000; Pierce). After electrophoresis separation, membrane transformation, antibody incubation, and chemiluminescence imaging, the bands were obtained and densitometric analysis was performed using ImageJ. All assays were repeated three or more times.

### pH curve assay

The stains were cultured in macrophage-mimicking liquid medium (pH 5; 0.17% YNB+2% CAA+0.2% glucose) [23,26] or CAA liquid medium (0.17% YNB+2% CAA) [32] at an initial concentration of 2 × 10^6^ CFU/ml at 30°C with shaking. After 0, 2, 4, 8, 12, 24, 48 and 72 h, 4 ml of the suspension was pipetted into a 12-well plate, and the pH value was detected by a pH metre (Mettler Toledo). All assays were repeated three or more times.

### Bromocresol purple (BCP) colorimetric pH assay

BCP, a pH indicator with a colorimetric pH range of approximately 5.2 (yellow) to 6.8 (purple) [25], was added to macrophage-mimicking solid medium (pH 5; 0.17% YNB+2% CAA+0.2% glucose+2% agar) or CAA solid medium (0.17% YNB+2% CAA+2% agar). To every 2 ml of solid medium, 2 μl of BCP (0.01% vol/vol) [26,27] was added and pipetted into a 12-well plate. Five microlitres of *C. albicans* cells (2 × 10^6^ CFU/ml) were seeded into the plate. The control well is a blank well. After 48 h of culture at 37 °C, the colour changes of the medium were observed and compared with the BCP colorimetric pH scale. All assays were repeated three or more times.

### Filamentation assays

Before imaging under an inverted microscope (Leica, DMi8), a 2 × 10^5^ CFU/ml suspension of strains in macrophage-mimicking liquid medium, macrophage-mimicking liquid medium supplemented with cAMP (1, 5, or 10 mM; MCE, CAS: 60-92-4), macrophage-mimicking liquid medium with different pH values (adjusted to 4.0, 6.0, 7.0, 8.0) or CAA liquid medium was pipetted into sterile 12-well plates and then incubated at 37°C for 4 h. Additionally, 5 μl of strains (2 × 10^6^ CFU/ml) resuspended with PBS were seeded on macrophage-mimicking solid medium or CAA solid medium and were then stayed at 37°C for 7 d. All assays were repeated three or more times.

### Growth curves and cell viability assays

To assess the cell growth, the strains were seeded in CAA liquid medium at an initial concentration of 2 × 10^6^ CFU/ml and cultured at 30°C, then the OD_600_ was evaluated at intervals. Then, to assess the cells viability, the strains were cultured in the above medium, and 100 μl of the suspension was removed at intervals for the endpoint dilution assay [38]. All assays were repeated three or more times.

### Proteomic analysis

The proteins extracted from the strains by liquid nitrogen grinding method were enzymolized twice by trypsin to obtain the peptide. Then, the obtained peptides were desalted, labelled using a TMT kit/iTRAQ kit (Thermo Scientific), separated by high performance liquid chromatography, then assessed by tandem mass spectrometry (MS/MS) (Thermo Scientific). Finally, database search and quality control of the obtained data were carried out. All assays were repeated three or more times.

### Seahorse XFe96 assay

To evaluate OXPHOS function in *C. albicans*, the mitochondrial basal oxygen consumption rate (OCR) was detected with a kit for seahorse XFe96 assay (Agilent) [45]. Strains were seeded in 0.03% poly-L-lysine pretreated microplates (5 × 10^5^ cells/well) and cultured at 30°C without CO_2_ for 1 h. During sequential addition of dicyclohexylcarbodiimide (DCCD), carbonyl cyanide 4-(trifluoromethoxy) phenylhydrazone (FCCP) and rotenone (Rot)/antimycin A (AA), the OCR was monitored by an instrument (Agilent) and analysed by Wave 2.6 software. The values of basal respiration and ATP produced by OXPHOS representative of mitochondrial function were calculated from the above available data. All assays were repeated three or more times.

### Statistical analysis

Unless otherwise stated, all statistical data were obtained from three or more independent trials and presented as means ± standard deviation (SD). The different types of data were analysed by the log-rank tests, the two-tailed unpaired Student’s *t* test and the one-way ANOVA, respectively. And the above processes were carried out by GraphPad Prism software (version 8.0). In general, *P* > 0.05 means statistically significant, while in the proteomic analysis, a fold change >1.5 means statistically significant (*P* > 0.05). All assays were repeated three or more times.

## Results

### Deletion of the F_1_F_o_-ATP synthase α subunit abrogates host inflammatory responses

**Figure 1.**
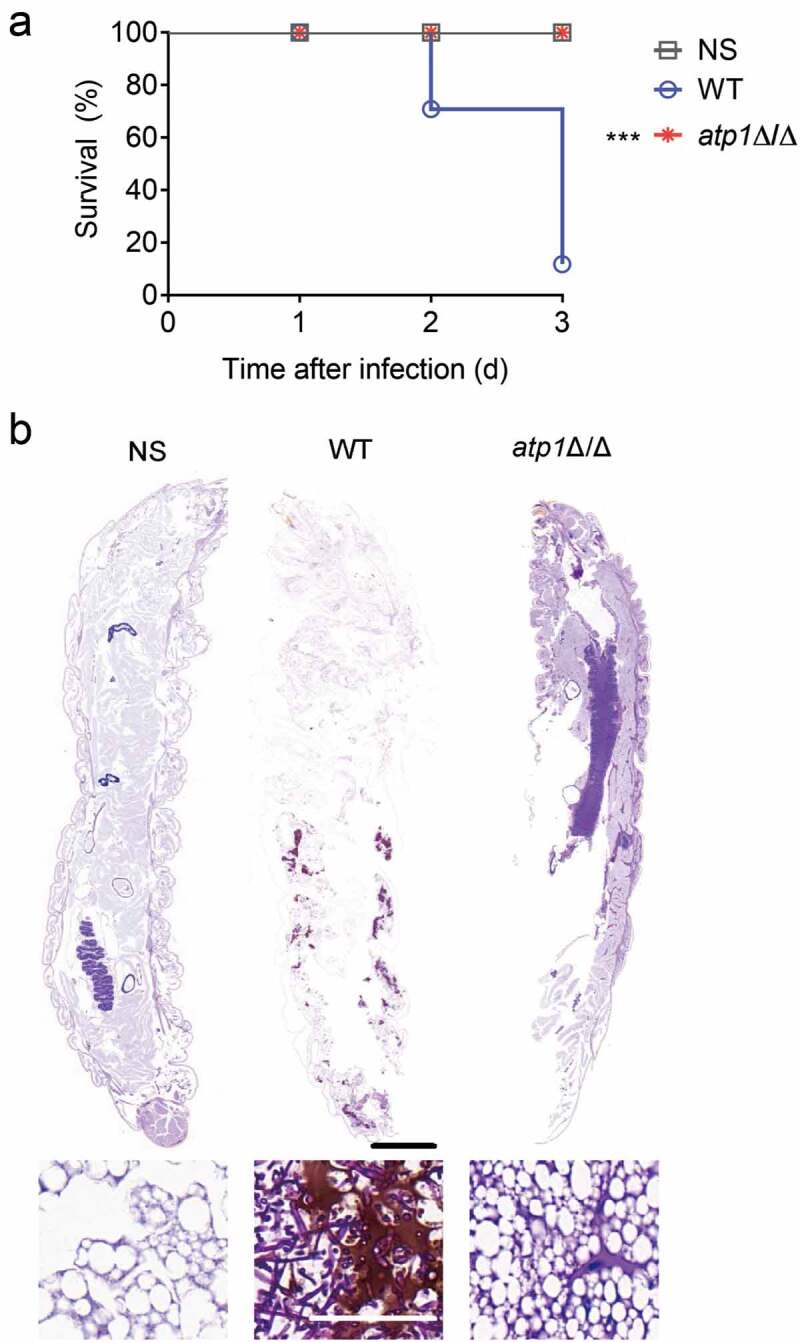
Deletion of the F_1_F_o_-ATP synthase α subunit abrogates the inflammatory response in *G. mellonella*. (a) After injection of 100 μl NS, WT or *atp1*δ/Δ (5 × 10^5^ CFU per larva), the survival curves of *G. mellonella* (*n* = 12) were obtained and analysed by log-rank tests. ***P < 0.001. (b) Seventy-two hours after injection of 100 μl NS, WT or *atp1*δ/Δ (5 × 10^5^ CFU per larva), the sections of *G. mellonella* (*n* = 3) were stained by PAS. Scale bars, 2000 µm (top), 50 µm (bottom). These images represent the results of one of three separate experiments.

Sepsis is characterized by dysregulation and overactivation of host-protective innate immunity [46], with early manifestations dominated by hyperinflammatory response [12]. *G. mellonella* has a single innate immune system that is highly similar to the mammalian innate immune system [40,41] and could provide a specifical observation of the innate immune response that dominates the septic hyperinflammatory response, and an amino acid-rich haemolymph [40] that might provide an amino acid-rich host niche for observing the phenotype of *C. albicans* strains. Therefore, for the purpose of studying the potential influence of the F_1_F_o_-ATP synthase α subunit of *C. albicans* on the septic hyperinflammatory response of host, the *G. mellonella* model was used in this study. We challenged *G. mellonella* with a reported lethal dose of *C. albicans* [42] and observed that the survival rate of *G. mellonella* infected with *atp1*Δ/Δ was 100%, while that of WT was 16.6% (Figure 1a). Histopathologic analysis showed that *G. mellonella* infected with *atp1*Δ/Δ had no abnormal morphology, tissue injury, hyphae, pseudohyphae, yeasts or inflammatory cells aggregation, which was the same as that in the NS control group (Figure 1b). In contrast, *G. mellonella* infected with WT exhibited a visible tissue injury with masses of hyphae, the crucial pathogenic forms in the induction of inflammatory responses, as well as numerous inflammatory cells (Figure 1b). The above results reveal that F_1_F_o_-ATP synthase α subunit deletion eliminates the fatal inflammatory response in *G. mellonella*.

**Figure 2.**
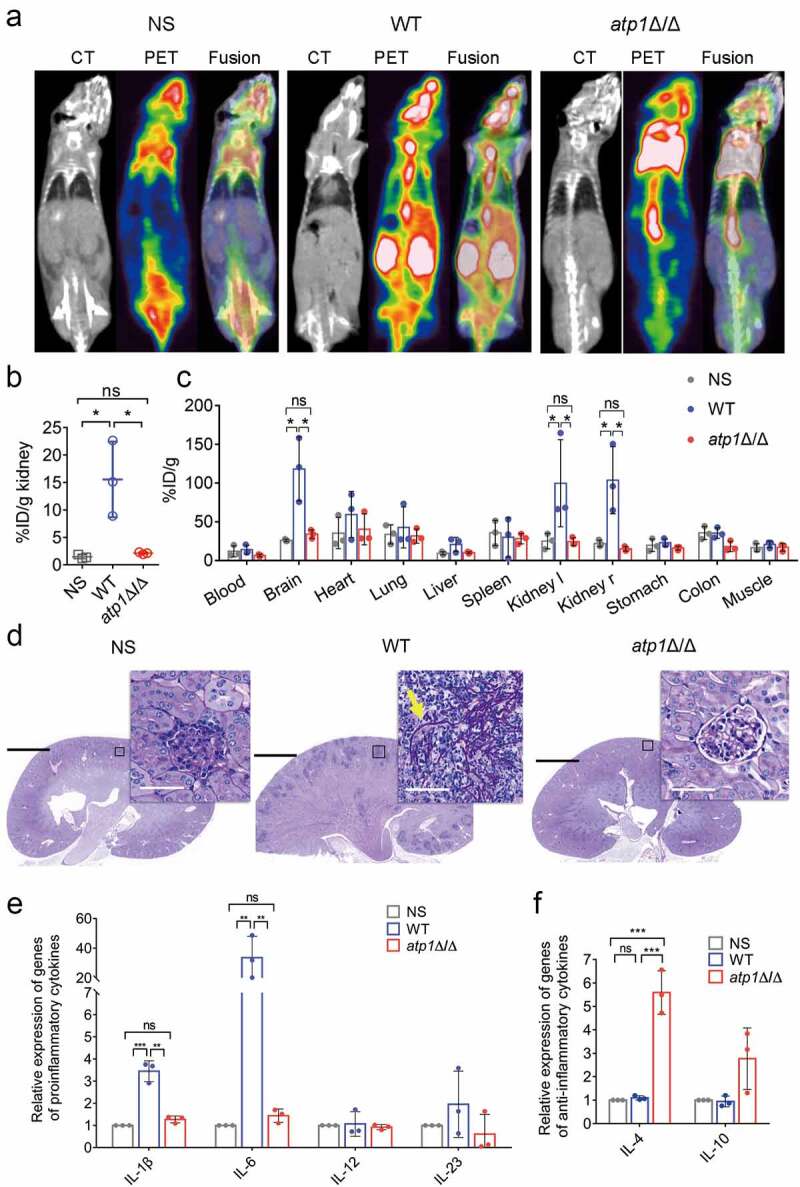
Deletion of the F_1_F_o_-ATP synthase α subunit eliminates the inflammatory response in mice. (A-F) Seventy-two hours after intravenous injection of 100 μl NS, WT or *atp1*δ/Δ (5 × 10^5^ CFU per mouse), mice (*n* = 3) in each group were subjected to the following experiments. (a) the microPET/CT imaging of mice (*n* = 3) was performed using a microPET/CT scanner. (b) the intensity of PET signal in the kidneys of mice (*n* = 3) was quantitatively analysed. (c) the distribution of [^18^F]FDG in the *ex vivo* organs obtained from mice (*n* = 3) was assessed by using a γ-counter. The left kidney and right kidney were represented by Kidney l and Kidney r, respectively. (d) the sections of kidneys of mice (*n* = 3) were stained by PAS. The scale bars are 2000 µm and 50 µm in sections and inserted images, respectively. (e) the mRNA expression levels of the proinflammatory cytokines IL-1β, IL-6, IL-12 and IL-23 in the kidneys of mice (*n* = 3). (f) the mRNA expression levels of the anti-inflammatory cytokines IL-4 and IL-10 in the kidneys of mice (*n* = 3). (a and d) These images represent the results of one of three separate experiments. (b, c, e and F) the results from three separate trials were analysed by one-way ANOVA and represented by the means ± SD. *P < 0.05, **P < 0.01; ***P < 0.001; ns, not significant.

To further reveal the important impact of the F_1_F_o_-ATP synthase α subunit on host inflammatory responses, according to the fact that the uptake and accumulation of [^18^F]FDG in active inflammatory cells was obviously higher than that in normal cells [47], microPET/CT combined with radioactivity measurement by γ-counting [48] was used to monitor the inflammatory response in tissues and organs – especially the kidney, the target organ of IC [17]—in infected mice. The microPET/CT imaging displayed that the uptake of [^18^F]FDG in the systemic organs of *atp1*Δ/Δ-infected mice had no difference from that in NS-control mice, while was obviously decreased compared with that in WT-infected mice (Figure 2a). Consistent with the findings of microPET/CT imaging, the intensity of the PET signal in the kidneys of *atp1*Δ/Δ-infected mice was similar to that in NS-control mice but visibly weaker than that in WT-infected mice (*P* < 0.05) (Figure 2b). To more precisely estimate the scope of the inflammatory responses, a γ-counter was used to assess the distribution of [^18^F]FDG in *ex vivo* organs, and the release of γ-rays in the kidneys, livers, spleens and brains of *atp1*Δ/Δ-infected mice was similar to that in NS-control mice but dramatically lower than that in WT-infected mice, particularly in the kidneys and brains (*P* < 0.05) (Figure 2c). Consistent with the microPET/CT results, histopathologic analysis showed that the kidneys of mice infected with *atp1*Δ/Δ and treated with NS were similar in appearance, which displayed a normal morphology without tissue damage, infiltration of hyphae, pseudohyphae, yeast cells or inflammatory cells (Figure 2d). In contrast, WT-infected mice exhibited an obvious renal tissue injury with infiltration and accumulation of hyphal masses in the renal cortex, renal tubules and renal pelvis, accompanied by numerous inflammatory cells infiltration, mainly neutrophils (Figure 2d). In short, the above results reveal that F_1_F_o_-ATP synthase α subunit deletion leads to the inability of *C. albicans* to induce inflammatory responses in systemic organs.

To investigate the influence of the F_1_F_o_-ATP synthase α subunit on inflammatory cytokine production, the mRNA expression levels of the proinflammatory cytokines IL-1β, IL-6, IL-12 and IL-23 and the anti-inflammatory cytokines IL-4 and IL-10 in the kidneys of infected mice was measured by RT‐qPCR. The mRNA expression levels of IL-1β and IL-6, the critical proinflammatory cytokines that can be induced by hyphae [7,11], in the kidneys of *atp1*Δ/Δ-infected mice were not distinguish from those in the kidneys of NS-control mice but were significantly lower than those in the kidneys of WT-infected mice (*P* < 0.01) (Figure 2e). Moreover, the mRNA expression level of the anti-inflammatory cytokine IL-4, which can control the progression of sepsis [19], in kidneys of *atp1*Δ/Δ-infected mice was significantly higher than that in the kidneys of WT-infected mice and NS-control mice (*P* < 0.001) (Figure 2f). The above results clarify that the deletion of F_1_F_o_-ATP synthase α subunit fails to elicit an excessive transcription of IL-1β and IL-6 and upregulates the transcription of IL-4, resulting to the abrogation of the inflammatory response.

### Deletion of the F_1_F_o_-ATP synthase α subunit prevents C. albicans from escaping macrophages

**Figure 3.**
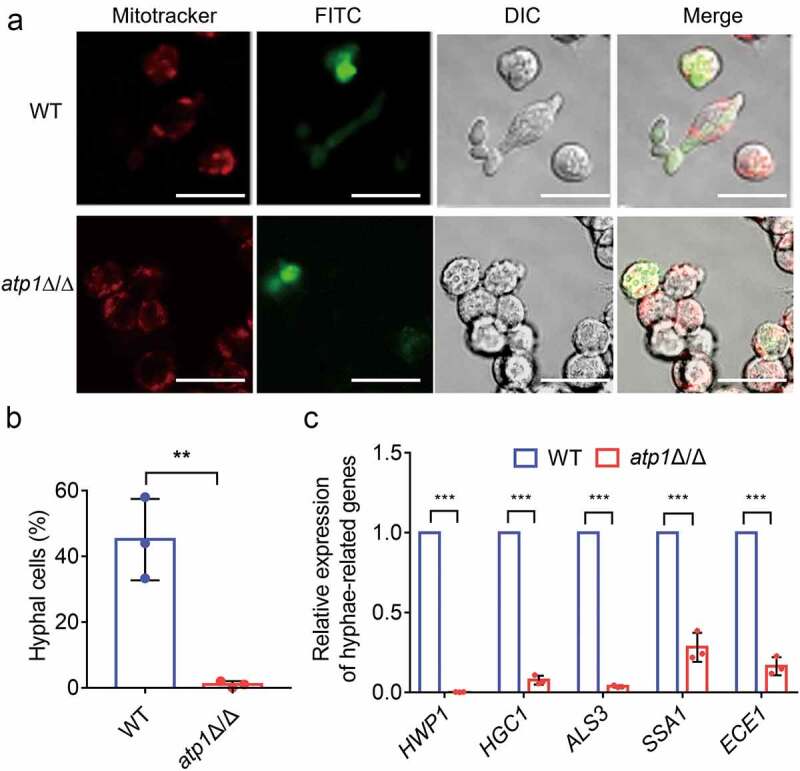
Deletion of the F_1_F_o_-ATP synthase α subunit inhibits *C. albicans* escaping from macrophage. (a) the CLSM imaging of pretreated RAW264.7 macrophages (red) and FITC-stained WT or *atp1*δ/Δ cells (green) after 3 h co-culture at a 1:1 ratio. The channel settings were E x 644/Em655 (red), E x 488/Em525 (green), and DIC, respectively. Scale bar, 20 µm. These images represent the results of one of three separate experiments. (b) the percentages of WT and *atp1*δ/Δ hyphal cells inside macrophages were calculated using ImageJ. (c) the mRNA expression levels of the virulence-related genes *HWP1*, *HGC1*, *ALS3*, *SSA1* and *ECE1* in WT and *atp1*δ/Δ by the cocultured with macrophages. (b and c) the results from three separate trials were analysed by two-tailed unpaired Student’s *t* test and represented by the mean ± SD. **P < 0.01, ***P < 0.001.

Macrophages are critical for controlling host inflammatory responses by phagocytizing and killing *C. albicans* [14,22]. Considering that filamentation is a crucial virulence factor in the process of *C. albicans* escaping from macrophages and the subsequent induction of inflammatory responses [14]. Therefore, to observe the effect of F_1_F_o_-ATP synthase α subunit deletion on the filamentation of *C. albicans* after macrophages phagocytizing *C. albicans*, a *C. albicans*-macrophage co-culture model was established. As observed by CLSM, WT formed hyphae to destroy and escape from macrophages, while *atp1*Δ/Δ was trapped inside macrophages only in yeast form after phagocytosis (Figure 3a). Similarly, the proportion of *atp1*Δ/Δ hyphal cells inside macrophages was visibly lower than that of WT hyphal cells (*P* < 0.01) (Figure 3b). Consistent with this finding, RT‐qPCR indicated that the mRNA expression levels of virulence-related genes (*HWP1*, *HGC1*, *ALS3*, *SSA1* and *ECE1*) in *atp1*Δ/Δ were dramatically downregulated compared with those in WT (*P* < 0.001) (Figure 3c). These results indicate that F_1_F_o_-ATP synthase α subunit deletion causes filamentation defects of *C. albicans* inside macrophages that prevents *C. albicans* from escaping macrophages.

### The F_1_F_o_-ATP synthase α subunit activates cAMP-PKA pathway-induced filamentation by alkalinizing environment

**Figure 4.**
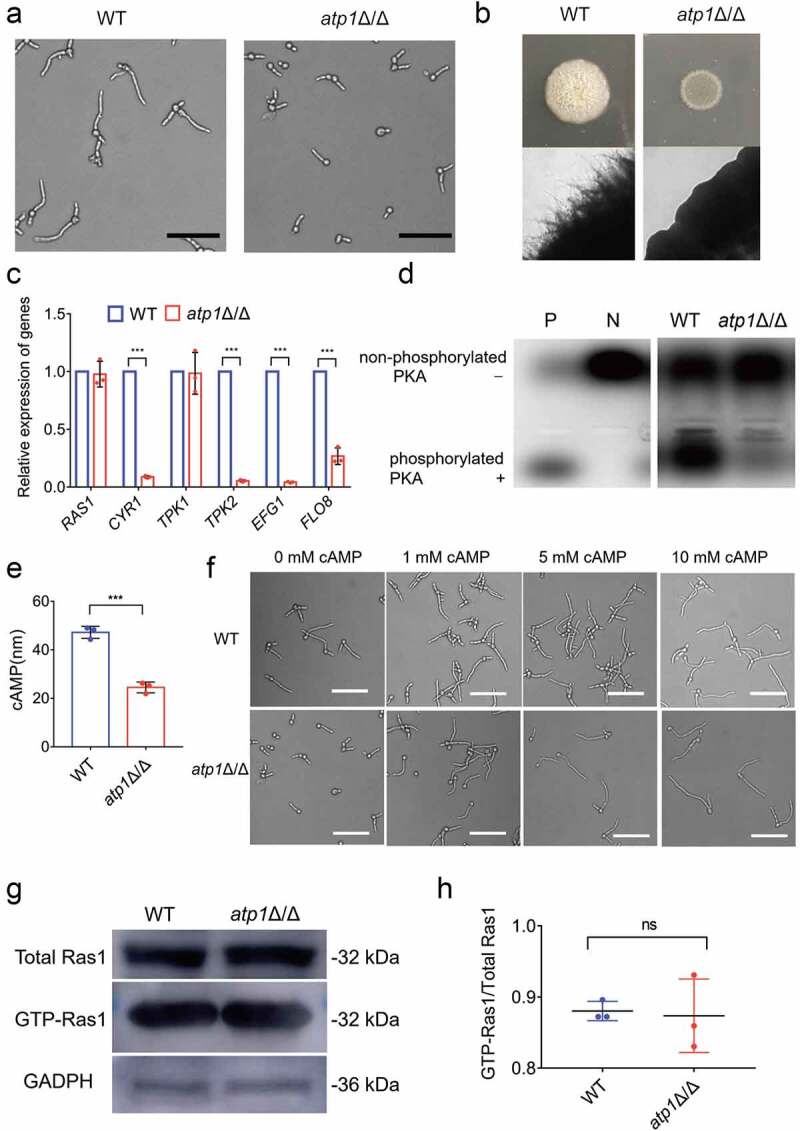
Deletion of the F_1_F_o_-ATP synthase α subunit inhibits Ras1-independent cAMP-PKA pathway-induced filamentation. a and b) Filamentation of WT and *atp1*δ/Δ was induced at 37 °c (a) in macrophage-mimicking liquid medium (pH 5; 0.17% YNB+2% CAA+0.2% glucose) for 4 h and (b) on solid medium (pH 5; 0.17% YNB+2% CAA+0.2% glucose+2% agar) for 7 d, respectively. (c) the mRNA expression levels of the cAMP/PKA pathway genes *RAS1*, *CYR1*, *TPK1*, *TPK2*, *EFG1* and *FLO8* in WT and *atp1*δ/Δ were assessed by RT‐qPCR. (d) the phosphorylation of PKA in WT and *atp1*δ/Δ was assayed with a kit for cAMP-dependent phosphorylation of PKA. By comparison with a positive (P) control well and a negative (N) control well, ”+” and “-” indicate phosphorylated PKA and non-phosphorylated PKA, respectively. (e) the intracellular cAMP contents in WT and *atp1*δ/Δ were assessed with a kit for intracellular cAMP content assay. (f) Filamentation of WT and *atp1*δ/Δ in macrophage-mimicking medium was induced by adding exogenous cAMP (0, 1, 5, 10 mM) at 37 °C for 4 h. (g) the GTP-Ras1 (active Ras1, 32 kDa) levels in WT and *atp1*δ/Δ were determined with a Ras pull-down assay kit combined with an immunoblotting assay. GAPDH (36 kDa) acted as a loading control. (h) the ratio of band density of GTP-Ras1 to that of total Ras1 in WT and *atp1*δ/Δ was calculated using ImageJ. (a and f) Scale bar, 50 μm. (a, b, d, f and g) These images represent the results of one of three separate experiments. (c, e and h)The results from three separate trials were analysed by two-tailed unpaired Student’s *t* test and presented as the mean ± SD. ***P < 0.001; ns, not significant.

To reveal the regulatory mechanism by which the F_1_F_o_-ATP synthase α subunit affects filamentation of *C. albicans* inside macrophages, based on the reported characteristics of the acidic [23] and amino acid-rich [24,25] microenvironment inside macrophages, we established a macrophage-mimicking microenvironment model and designed experiments for investigation at different levels. Consistent with the results in the *C. albicans*-macrophage co-culture model, *atp1*Δ/Δ displayed marked filamentation defects in macrophage-mimicking liquid and solid media compared to that of WT (Figure 4a,b). RT‐qPCR indicated that the mRNA expressions of encoding-genes of important proteins Cyr1 [30,33] and Tpk2 [30] in the cAMP/PKA pathway, the core signalling pathway regulating filamentation in *C. albicans* [30], and the transcription factors Efg1 and Flo8 [30,33], located in the downstream of this pathway, were all notably decreased in *atp1*Δ/Δ compared with those in WT (*P* < 0.001) (Figure 4c). In addition, the level of phosphorylated PKA (active PKA) [30], which is necessary for the activation of Efg1 and Flo8, was lower in *atp1*Δ/Δ than that in WT (Figure 4d). Furthermore, the intracellular cAMP content, which determines the activation of PKA [30], was markedly lower in *atp1*Δ/Δ than that in WT (*P* < 0.001) (Figure 4e). Subsequently, the addition of exogenous cAMP caused a concentration-dependent restoration of *atp1*Δ/Δ filamentation, eventually reaching the WT level (Figure 4f). In addition, consistent with RT-qPCR results showing no difference in the mRNA levels of coding-gene of Ras1, an upstream intracellular signalling protein of the cAMP/PKA pathway [33], between *atp1*Δ/Δ and WT (Figure 4c), a Ras pull-down assay combined with immunoblotting showed that Ras1 activity (GTP-Ras1) was not significantly different between *atp1*Δ/Δ and WT (Figure 4g,h). These results reveal that F_1_F_o_-ATP synthase α subunit deletion inhibits filamentation by blocking the Ras1-independent cAMP/PKA pathway in a macrophage-mimicking environment.

**Figure 5.**
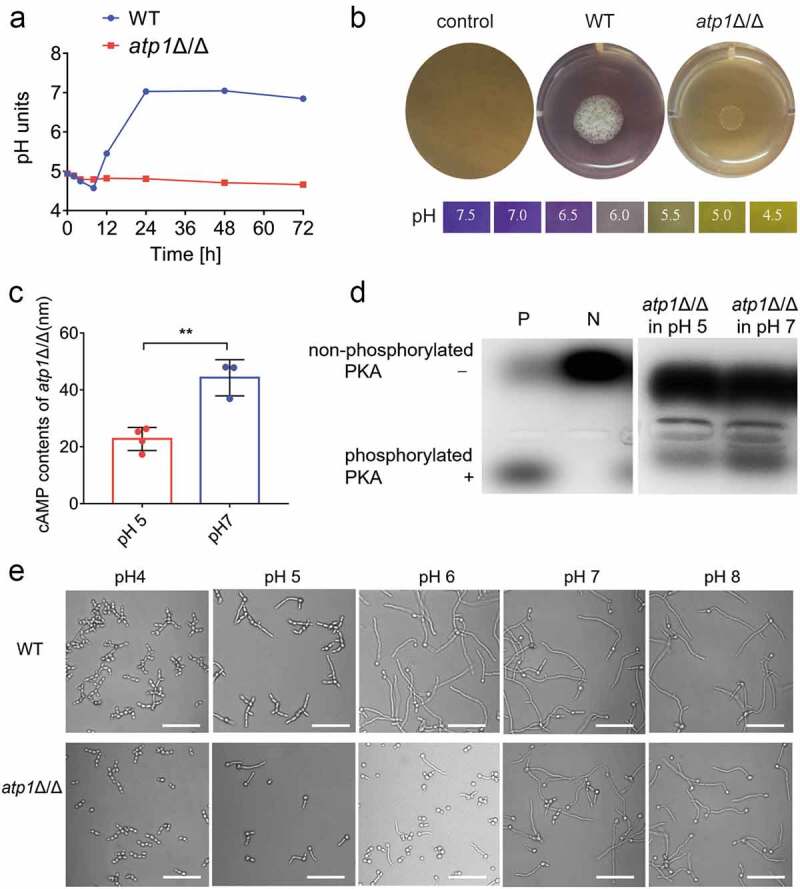
The F_1_F_o_-ATP synthase α subunit activates cAMP-PKA pathway-induced filamentation by alkalinizing environment. (a) the pH curves of WT and *atp1*δ/Δ cultured in macrophage-mimicking liquid medium (pH 5; 0.17% YNB+2% CAA+0.2% glucose) at 30 °C, 150 rpm. (b) the changes in the environmental pH of WT and *atp1*δ/Δ cultured in macrophage-mimicking solid medium (pH 5; 0.17% YNB+2%CAA+0.2% glucose+2% agar) supplemented with 0.01% (vol/vol) BCP at 37 °C for 2 d. (c) the intracellular cAMP contents in *atp1*δ/Δ cultured in pH 5 and pH 7 medium (0.17% YNB+2% CAA+0.2% glucose) were assessed with a kit for intracellular cAMP content assay. The results from three separate trials were analysed by two-tailed unpaired Student’s *t* test and represented by the mean ± SD. **P < 0.01. (d) the phosphorylation of PKA in *atp1*δ/Δ incubated in pH 5 and pH 7 media (0.17% YNB+2% CAA+0.2% glucose) was assayed with a kit for cAMP-dependent phosphorylation of PKA. By comparison with a positive (P) control well and a negative (N) control well, ”+” and “-” indicate phosphorylated PKA and non-phosphorylated PKA, respectively. (e) Filamentation of WT and *atp1*δ/Δ in pH 4, 5, 6, 7 and 8 media (0.17% YNB+2% CAA+0.2% glucose) after incubation at 37 °C for 4 h. Scale bar, 50 μm. (a, b, d, and e) These images represent the results of one of three separate experiments.

pH is one of the extracellular signalling factors acting upstream of the cAMP/PKA signalling pathway [31]. The pH curve displayed that the WT raised the pH of the liquid medium from 4.94 to 7.03 within 24 h (Figure 5a). Similarly, a BCP colorimetric pH assay showed that the WT raised the pH of the solid medium from 5.0 to 7.0 within 48 h (Figure 5b). In contrast, *atp1*Δ/Δ failed to raise the pH of either the liquid or solid medium (Figure 5a,b), indicating that the F_1_F_o_-ATP synthase α subunit is necessary for *C. albicans* to alkalinize environment. We next sought to determine whether the F_1_F_o_-ATP synthase α subunit activates the cAMP/PKA pathway to induce filamentation by alkalinizing environment. The environmental pH was raised by exogenous treatment from acidic (5.0) to neutral (7.0), and the cAMP content was increased and PKA activity was enhanced in *atp1*Δ/Δ accordingly (Figure 5c,d). In addition, the filamentation of *atp1*Δ/Δ was restored in a pH-dependent way to the degree exhibited in WT (Figure 5e). In short, the above results reveal that the F_1_F_o_-ATP synthase α subunit activates the cAMP/PKA pathway to induce filamentation by alkalinizing environment.

### Deletion of the F_1_F_o_-ATP synthase α subunit inhibits amino acid catabolism

**Figure 6.**
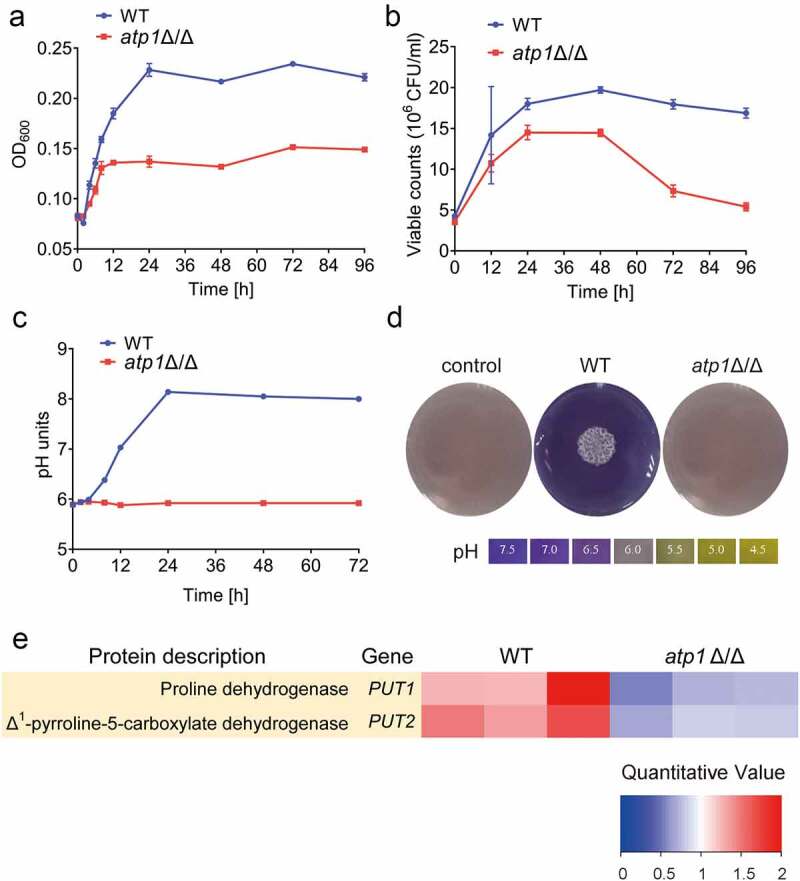
Deletion of the F_1_F_o_-ATP synthase α subunit fails to alkalinize environment by amino acid catabolism. (a) the cell growth of WT and *atp1*δ/Δ incubated in CAA liquid medium (0.17% YNB+2% CAA) over times was assessed by the cell density (OD_600_). (b) the cell viability of WT and *atp1*δ/Δ incubated in CAA liquid medium over times was determined by the viable cell count (10^6^ CFU/ml). (c) the pH curves of WT and *atp1*δ/Δ incubated in CAA liquid medium at 30 °C, 150 rpm. (d) the changes in the environmental pH of WT and *atp1*δ/Δ incubated on CAA solid medium supplemented with 0.01% (vol/vol) BCP at 37 °C for 2 d. These images represent the results of one of three separate experiments. (e) the protein expression levels of put1 and put2 were assessed using proteomic analysis. Three bars of each group indicate the results of three separate experiments. A fold change>1.5 means statistically significant (*P* < 0.05).

Phagocytized *C. albicans* produces NH_3_ through amino acid catabolism to neutralize the acidity of phagosomes, thereby inducing filamentation to destroy and escape from macrophages [26,27]. To explore the influence of the F_1_F_o_-ATP synthase α subunit on amino acid catabolism, we used CAA medium to provide a rich source of amino acids for *C. albicans* [26,27,32]. The growth curve showed that the cell growth of *atp1*Δ/Δ was dramatically inhibited compared with that of WT, showing decreases in the early growth phase, stationary phase and maximum growth rate (Figure 6a). In addition, the cell viability curve disclosed that the viability of *atp1*Δ/Δ cells was obviously lower than that of WT cells (Figure 6b). In addition, the pH curve showed that WT raised the pH of the liquid medium from 5.89 to 8.14 within 24 h (Figure 6c). Similarly, the BCP colorimetric pH assay showed that WT raised the pH of solid medium from the initial 6.0 to 7.5 within 48 h (Figure 6d). However, *atp1*Δ/Δ failed to raise the pH of either the liquid or solid medium (Figure 6c,d). These results reveal that the failure of the F_1_F_o_-ATP synthase α subunit deletion mutant to alkalinize environment is due to impaired amino acid catabolism. Put1 and Put2 are two essential enzymes in amino acid catabolism in *C. albicans* to alkalinize environment [33]. Proteomic analysis displayed that the protein expression levels of Put1 and Put2 in *atp1*Δ/Δ were observably decreased compared with those in WT (*P* < 0.05) (Figure 6e), clarifying that F_1_F_o_-ATP synthase α subunit deletion inhibits amino acid catabolism by downregulating Put1 and Put2.

### Deletion of the F_1_F_o_-ATP synthase α subunit suppresses OXPHOS

**Figure 7.**
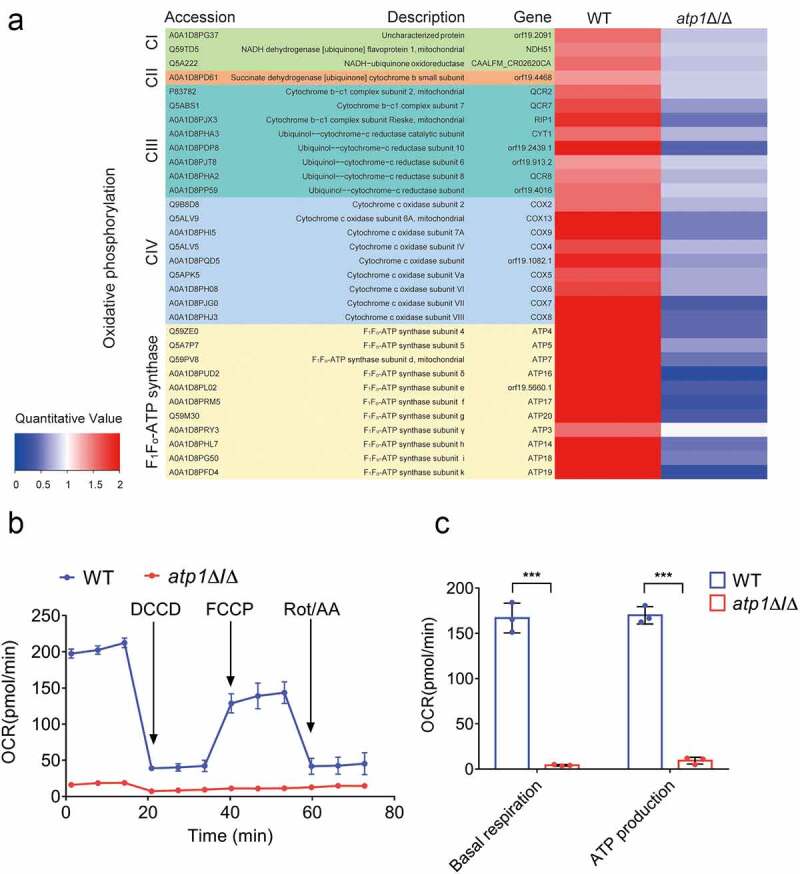
Deletion of the F_1_F_o_-ATP synthase α subunit severely impairs OXPHOS. (a) the expression levels of etc proteins were determined using proteomic analysis. The bars of each group indicate the results of three separate experiments. A fold change>1.5 means statistically significant (*P* < 0.05). (b) After adding DCCD, FCCP and Rot/AA in sequence, the OCR of WT and *atp1*δ/Δ were evaluated. (c) the basal respiration and ATP produced by OXPHOS were calculated. The results from three separate trials were analysed by two-tailed unpaired student’s *t* test and presented as the mean ± SD. ***P < 0.001.

The functional ETC, whose terminal and key enzyme is F_1_F_o_-ATP synthase [37], is required for Put1 activity [35,36]. Additionally, OXPHOS is required for *C. albicans* to alkalinize environment via amino acid catabolism [32]. To determine the effect of the F_1_F_o_-ATP synthase α subunit on the ETC proteins involved in OXPHOS, we performed proteomic analysis, which showed that compared with WT, *atp1*Δ/Δ exhibited a significant downregulation of the expression of most proteins consisted in complex I (CI), complex II (CII), complex III (CIII), complex IV (CIV) and F_1_F_o_-ATP synthase (complex V, CV) (*P* < 0.05) (Figure 7a). To explore the influence of the F_1_F_o_-ATP synthase α subunit on OXPHOS function, a Seahorse XFe96 assay was performed. The OCR of *atp1*Δ/Δ was dramatically lower than that of WT (Figure 7b). Further calculation revealed that the basal respiration rate of *atp1*Δ/Δ was markedly reduced compared with that of WT and that the amount of ATP produced by OXPHOS was approximately 5% of that produced in WT (Figure 7c), suggesting that OXPHOS function was severely suppressed. These results imply that F_1_F_o_-ATP synthase α subunit deletion severely suppresses OXPHOS.

## Discussion

Sepsis, characterized by an early hyperinflammatory response [6,12], is an important ultimate cause of fatality in individuals with invasive candidiasis [2,4,5]. Here, we eluminate the *C. albicans* F_1_F_o_-ATP synthase α subunit induces host inflammatory responses by controlling its own amino acid catabolism.

*C. albicans* mutant with deleted F_1_F_o_-ATP synthase α subunit failed to induce an inflammatory response in infected *G. mellonella* and mice. Filamentation endows *C. albicans* with proinflammatory capability [7] to elicit the production and excretion of host proinflammatory cytokines such as interleukin (IL)-1β [7–10,49] and IL-6 [11] through recognition of hyphal pathogen-associated molecular patterns [9], secretion of hyphae-specific aspartyl proteinases (SAP4–6) [49,50] or hyphae-specific toxin candidalysin [7], thus inducing host inflammatory responses. Under continuous stimulation by *C. albicans*, the overwhelming and rapid release of proinflammatory cytokines is induced, and these cytokines enter the circulation to trigger a cytokine storm [6,13], causing a systemic lethal hyperinflammatory response [15,16]. As a result, a large number of inflammatory cells are recruited to infiltrate and damage systemic tissues and organs [13], and kidney damage is the most common and serious effect [15–17]. Compared with WT, *atp1*Δ/Δ abrogated the systemic inflammatory response in infected *G. mellonella* and mice, especially in the kidneys of infected mice, and no hyphal aggregation or inflammatory cell infiltration was found in the tissues. Moreover, in the kidneys of infected mice, *atp1*Δ/Δ failed to cause an excessive transcription of the hyphae-specific proinflammatory cytokines IL-1β and IL-6 [7,11] as the same as WT, and significantly upregulated the transcription of the anti-inflammatory cytokine IL-4 that can control the progression of sepsis [19] when compared to WT. Therefore, it is most likely due to the filamentation-associated defects that F_1_F_o_-ATP synthase α subunit deletion displays a failure of the excessive transcription of IL-1β and IL-6 and upregulates the transcription of IL-4, resulting to the abrogation of the inflammatory response.

Macrophages are particularly critical for controlling the renal inflammatory response induced by *C. albicans* [17,22]. Enhancement of macrophage phagocytosis and killing of *C. albicans* alleviates the hyperinflammatory response by reducing the overproduction of IL-1β [14]. Nutrient restriction and acidic phagosomes are two important intracellular anti-*Candida* mechanisms of macrophages [23]. However, amino acids, which are abundant inside macrophages, are an important alternative carbon source for *C. albicans* to escape macrophage killing [24–26]. *C. albicans* induces filamentation by neutralizing the acidity of phagosomes through amino acid catabolism [26,27]. Furthermore, filamentation enables *C. albicans* to kill and escape from macrophages through mechanical penetration of cell membranes [29], NLRP3/caspase-1 inflammasome-dependent pyroptosis [51], cytolysis [52] and glucose competition [53]. Subsequently, *C. albicans* that avoid timely killing and elimination by macrophages invade organs and tissues throughout the body through hyphal infiltration into vascular endothelial cells [16,54,55], leading to the induction of systemic inflammatory responses [15,16]. In contrast to WT, which damaged and escaped from macrophages by inducing filamentation, *atp1*Δ/Δ was trapped inside macrophages in yeast form, accompanied by a reduction in hyphal cells and downregulation of the transcription of hypha-related genes. This finding indicates that the filamentation defects of the F_1_F_o_-ATP synthase α subunit deletion mutant prevent its escape from and facilitate its elimination by macrophages; thus, this mutant fails to induce host inflammatory responses.

The infection advance of *C. albicans* to the host is determined by the joint action of growth ability and virulence factors [56], but not all factors play an equally important role in the pathogenic process. The normally growing nonfilamentous *C. albicans* mutants are avirulent [57]. Likewise, the cells of *C. albicans* mutant [57] locked in the yeast phase fail to elicit host inflammatory responses as the same as hyphal cells [8,10]. The *atp1*Δ/Δ mutant was unable to form hyphae to induce inflammatory response in the *G. mellonella* treated with a high dose of *C. albicans* cells (5 × 10^5^ CFU per larva [42]), and it also failed to induce excessive transcription of hyphae-specific proinflammatory cytokines [7,11] in the mouse kidneys, indicating that the elimination of host inflammatory response by deleting F_1_F_o_-ATP synthase α subunit is more likely due to filamentation-associated defects than to reduced growth ability. Furthermore, the cells of *C. albicans* mutant locked in the yeast phase are unable to escape from macrophages despite the number of yeast cells within the macrophage doubled within hours [24]. The *atp1*Δ/Δ mutant existed as yeast form inside macrophages, with a reduced hyphal cell formation rate and a decreasing expression of hypha-related pathogenic genes, indicating that the inability of the *atp1*Δ/Δ mutant to escape from macrophages is mainly due to the filamentation defects rather than the reduced growth ability. Overall, in the *atp1*Δ/Δ mutant, filamentation-associated defects occupy an overwhelmingly critical role in dominating the elimination of host inflammatory responses and escaping from macrophages.

The cAMP/PKA pathway is the core signalling pathway regulating filamentation [30]. This pathway is controlled by many signalling molecules [30] and one of them is pH [31]. By mimicking the acidic [23] and amino acid-rich [25] microenvironment inside macrophages, it was found that the filamentation of *atp1*Δ/Δ was inhibited by blockade of the cAMP/PKA pathway. Furthermore, *atp1*Δ/Δ failed to alkalinize the acidic environment. Acidic pH downregulates Cyr1 activity by decreasing the intracellular bicarbonate pool, which in turn decreases cAMP production to inhibit filamentation [31]. Therefore, raising the ambient pH from acidic to neutral by exogenous treatment activated the cAMP/PKA pathway and induced the filamentation of *atp1*Δ/Δ, revealing that the F_1_F_o_-ATP synthase α subunit activates cAMP/PKA pathway-induced filamentation by alkalinizing environment.

Mitochondrial proline dehydrogenase (Put1) and ∆^1^-pyrroline-5-carboxylate dehydrogenase (Put2) are two essential enzymes of amino acid catabolism and their deletion dramatically prevents *C. albicans* from alkalizing environment [33]. Due to the downregulation of Put1 and Put2 protein expression in *atp1*Δ/Δ, this strain could not alkalinize environment via amino acid catabolism. Moreover, a functional electron transport chain (ETC) is required for Put1 activity [35,36], and respiratory deficient inhibits Put2 function [58]. In addition, inhibition of oxidative phosphorylation (OXPHOS) prevents *C. albicans* from catabolizing amino acids to alkalinize environment [32]. In *atp1*Δ/Δ, the expression of most ETC proteins involved in OXPHOS was significantly downregulated, and OXPHOS function was severely impaired. Therefore, it is speculated that the severe impairment of OXPHOS caused by F_1_F_o_-ATP synthase α deletion may cause inhibition of amino acid catabolism through downregulation of Put1 and Put2 protein expression.

**Figure 8.**
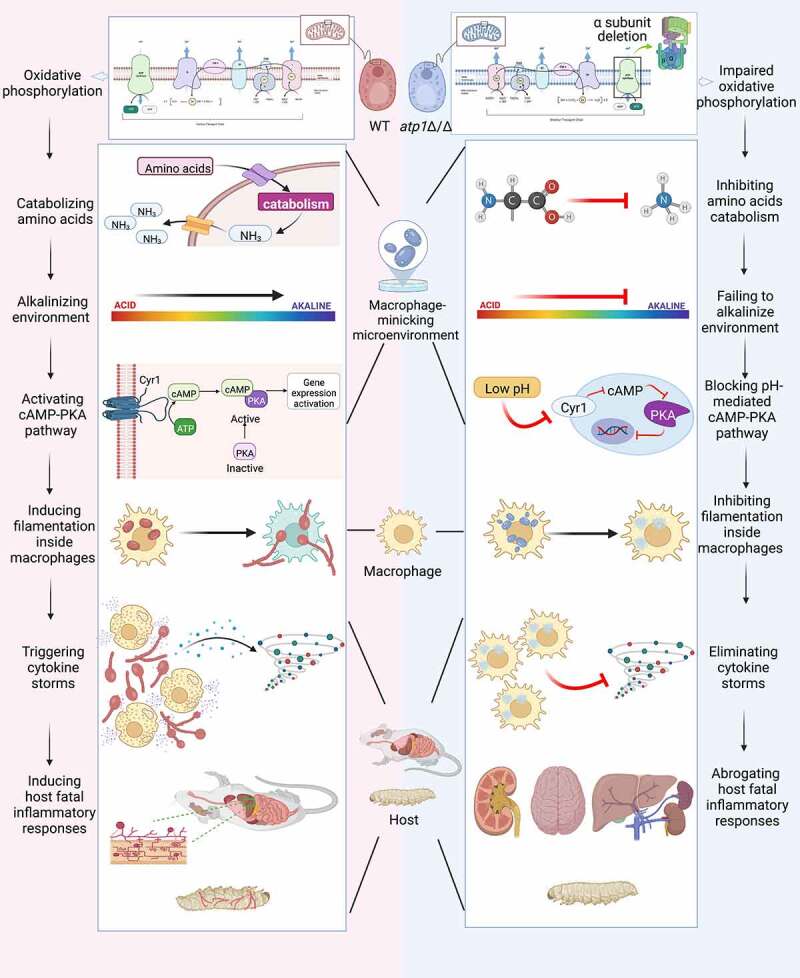
The F_1_F_o_-ATP synthase α subunit of *C. albicans* induces host inflammatory responses by controlling its own amino acid catabolism. Deletion of the F_1_F_o_-ATP synthase α subunit inhibits amino acid catabolism by impairing OXPHOS and then blocks cAMP/PKA pathway-induced filamentation due to failure to alkalinize environment, in turn preventing *C. albicans* from escaping macrophages and eventually abrogating host systemic inflammatory responses by abolishing the excessive transcription of IL-1β and IL-6 and upregulating the transcription of IL-4. Black arrows indicate facilitation, and red arrows indicate obstruction.

In summary, we reveal that deletion of the F_1_F_o_-ATP synthase α subunit of *C. albicans* inhibits amino acid catabolism by impairing OXPHOS and then blocks cAMP/PKA pathway-induced filamentation due to failure to alkalinize environment, in turn preventing *C. albicans* from escaping macrophages and eventually abrogating host systemic inflammatory responses by abolishing the excessive transcription of IL-1β and IL-6 and upregulating the transcription IL-4 (Figure 8). Therefore, the F_1_F_o_-ATP synthase α subunit of *C. albicans* induces host inflammatory responses by controlling its own amino acid catabolism. It is important to find drugs that inhibit F_1_F_o_-ATP synthase α subunit activity to control the induction of host inflammatory responses.

## Supplementary Material

Supplemental Material

## Data Availability

All data are available from the authors upon reasonable request.
