# Supplementary material for: The F1Fo-ATP synthase α subunit of Candida albicans induces inflammatory responses by controlling amino acid catabolism
Source: Virulence. 2023 Mar 29;14(1):2190645. doi: 10.1080/21505594.2023.2190645 (PMC10072111; doi:10.1080/21505594.2023.2190645)
Supplement: Supplemental Material [file KVIR_A_2190645_SM5943.docx]

**Supplementary Table 1.** Primers used in this study.

| Primer | Sequence (5'-3') |
| --- | --- |
| IL-1β-F | CAACCAACAAGTGATATTCTCCATG |
| IL-1β-R | GATCCACACTCTCCAGCTGCA |
| IL-6-F | GAGGATACCACTCCCAACAGACC |
| IL-6-R | AAGTGCATCATCGTTGTTCATACA |
| IL-12-F | GGAAGCACGGCAGCAGAATA |
| IL-12-R | AACTTGAGGGAGAAGTAGGAATGG |
| IL-23-F | GCAGATTCCAAGCCTCAGTC |
| IL-23-R | TTCAACATATGCAGGTCCCA |
| IL-4-F | GGTCTCAACCCCCAGCTAGT |
| IL-4-R | GCCGATGATCTCTCTCAAGTGAT |
| IL-10-F | CTTACTGACTGGCATGAGGATCA |
| IL-10-R | GCAGCTCTAGGAGCATGTGG |
| GADPH-F | ACCACAGTCCATGCCATCAC |
| GADPH-R | TCCACCACCCTGTTGCTGT |
| HWP1-F | TGTCTACACTACATTCTGTC |
| HWP1-R | AGGAATAGATGGTTGTGAAC |
| HGC1-F | GTATCGCTGGTTCTCGTGCT |
| HGC1-R | AGGTGTACCACTACCACCATT |
| ALS3-F | CTCATTACACCAACCATACA |
| ALS3-R | GGATTCTGTGGTTGTAGTAT |
| SSA1-F | ATTGCTGAAGGTTATTTGGGTTC |
| SSA1-R | GGTGGCTTGTCTTTGAGAATC |
| ECE1-F | CCAAGCACCTACTGTTCC |
| ECE1-R | GATACCAGCAACAACAGAAT |
| RAS1-F | ATCAAGATGGATTAGCATTGG |
| RAS1-R | TGTTGTTGCTGTTGTTGTTG |
| CYR1-F | AGAAAGAAGACGATGAAACAG |
| CYR1-R | AGGAGAACTAGAGGATGTAGAC |
| TPK1-F | AGAACTTGCCAACAAACAAC |
| TPK1-R | TTTCTTGGTCAAGGAAAGAC |
| TPK2-F | TTGTTGCCTGAACGTTCTAC |
| TPK2-R | CTACCATTGTGAACTGATCTC |
| EFG1-F | ACAACCTCAGCATTACAATG |
| EFG1-R | ATAGGTACTGCTTGTTGACC |
| FLO8-F | AGCAAATGACTAAGATGGCTG |
| FLO8-R | AGTCGGAATTACCAGTGTTTC |
| 18S-F | CGCAAGGCTGAAACTTAAAGG |
| 18S-R | AGCAGACAAATCACTCCACC |
